# Radiative controls by clouds and thermodynamics shape surface temperatures and turbulent fluxes over land

**DOI:** 10.1073/pnas.2220400120

**Published:** 2023-07-10

**Authors:** Sarosh Alam Ghausi, Yinglin Tian, Erwin Zehe, Axel Kleidon

**Affiliations:** ^a^Biospheric Theory and Modelling Group, Max Planck Institute for Biogeochemistry, Jena 07745, Germany; ^b^International Max Planck Research School for Global Biogeochemical Cycles, Jena 07745, Germany; ^c^Institute of Water Resources and River Basin Management, Department of Civil Engineering, Geo and Environmental Sciences, Karlsruhe Institute of Technology – KIT, 76131 Karlsruhe, Germany; ^d^State Key Laboratory of Hydroscience and Engineering, Key Laboratory of Hydrosphere Sciences of the Ministry of Water Resources, Department of Hydraulic Engineering, Tsinghua University, 100084 Beijing, China

**Keywords:** land–atmosphere interactions, radiation, thermodynamics, clouds

## Abstract

Land surface temperatures (LSTs) are strongly shaped by radiation but are modulated by turbulent fluxes and hydrologic cycling as the presence of water vapor in the atmosphere (clouds) and at the surface (evaporation) affects temperatures across regions. Here, we used a thermodynamic systems framework forced with independent observations to show that the climatological variations in LSTs across dry and humid regions are mainly mediated through radiative effects. We first show that the turbulent fluxes of sensible and latent heat are constrained by thermodynamics and the local radiative conditions. This constraint arises from the ability of radiative heating at the surface to perform work to maintain turbulent fluxes and sustain vertical mixing within the convective boundary layer. This implies that reduced evaporative cooling in dry regions is then compensated for by an increased sensible heat flux and buoyancy, which is consistent with observations. We show that the mean temperature variation across dry and humid regions is mainly controlled by clouds that reduce surface heating by solar radiation. Using satellite observations for cloudy and clear-sky conditions, we show that clouds cool the land surface over humid regions by up to 7 K, while in arid regions, this effect is absent due to the lack of clouds. We conclude that radiation and thermodynamic limits are the primary controls on LSTs and turbulent flux exchange which leads to an emergent simplicity in the observed climatological patterns within the complex climate system.

## Significance

Land surface temperatures are a key characteristic of climate. Yet, understanding the main factors that shape them remains challenging because of the apparent dependence on many factors, such as radiation, turbulence, water availability, and vegetation. We use a fundamental, physical approach starting with radiation as the main forcing and constraining turbulent fluxes by their ability to perform maximum work to generate convective motion. This approach works very well in predicting observed climatological variations in surface temperatures, showing that arid regions are typically warmer due to the stronger solar heating in the absence of clouds. The implication is that the climatological variations of surface temperatures are predominantly shaped by radiation, clouds, and thermodynamic limits.

Land surface temperature (LST) is one of the most significant climatological variables, shaping the physical environment of terrestrial ecosystems and being most strongly affected by global warming. Regional and seasonal variations are strongly modulated by both, atmospheric conditions, such as clouds, humidity, and heat transport (1–5), and land surface conditions, such as soil moisture, land cover, and vegetation type (6–12). An emergent simple feature of this variability is associated with aridity as dry regions and periods are typically associated with warmer temperatures (13, 14). On the one hand, it can be looked upon as a reflection of reduced evaporative cooling related to water limitation. On the other hand, these regions are also characterized by the absence of clouds, which enhances warming by altering the local radiative conditions. Alternatively, clouds cool the humid regions by reducing the solar absorption at the surface while the surface also cools by increased evaporation. While these two mechanisms are not entirely independent of each other (15–17) they do have a different impact on the surface energy budget of the region. Due to the highly coupled nature of the surface–atmosphere system (8, 18), it becomes almost impossible to separate the role of these effects. This leads to a key question: How much do soil water limitation and clouds affect surface temperatures across dry and humid regions?

To answer this question, we need to understand the impact of changes in radiative forcings on the turbulent flux exchange of sensible and latent heat between the surface and the atmosphere. However, these fluxes seem to be strongly coupled to highly heterogeneous land surface characteristics and appear unconstrained by the energy balance alone. With limited observations of land surface variables, they further remain uncertain in climate models and are generally described using a bulk aerodynamic approach and semiempirical parameterizations (19–21). Owing to this inherent complexity, there remains substantial intermodel disagreement and biases in their estimates (22–24). This further makes it difficult to separate the roles of evaporation, turbulent fluxes, and local radiative conditions in shaping surface temperatures.

**Fig. 1.**
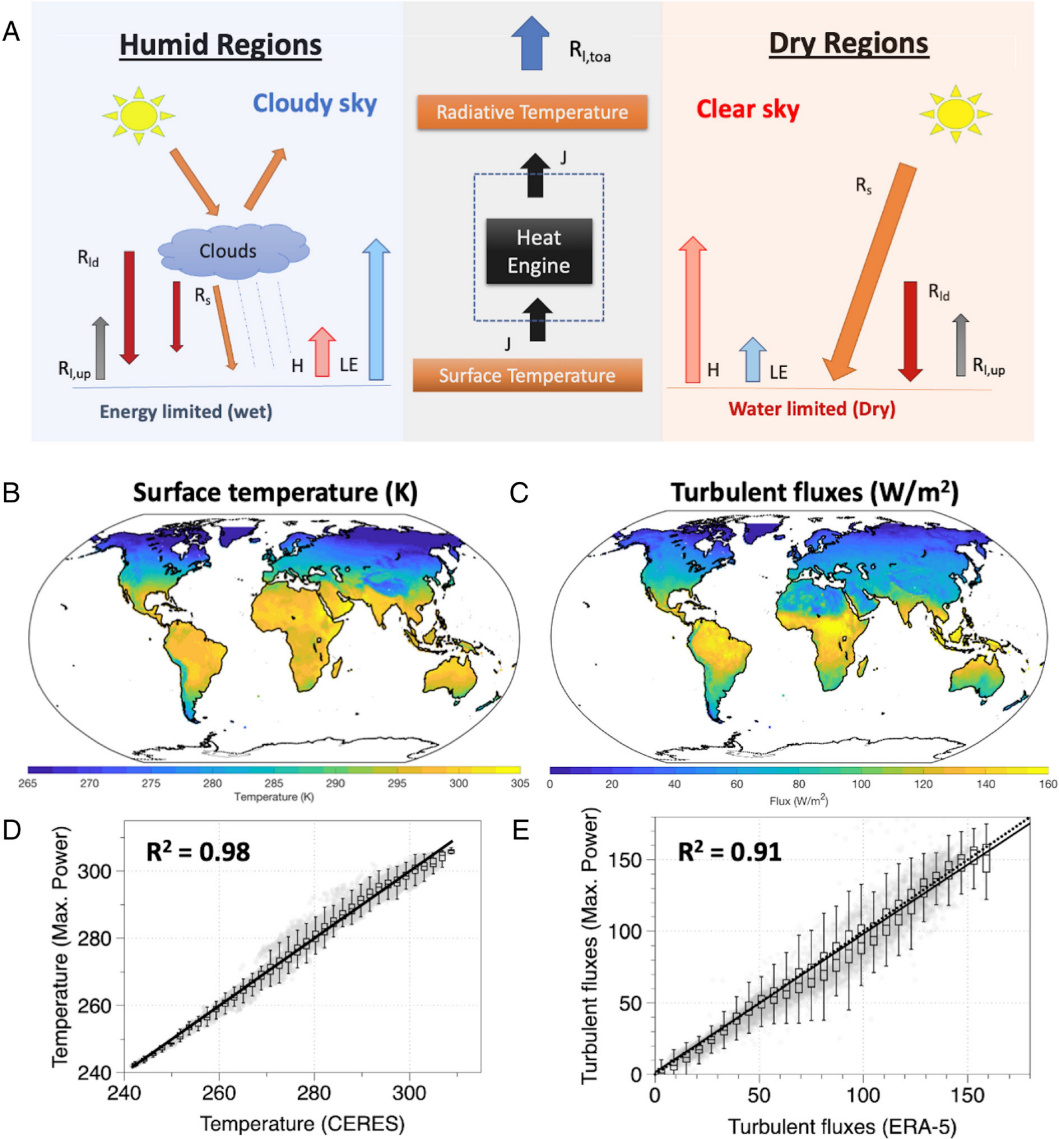
(*A*) Conceptual diagram of the surface–atmosphere system as an idealized heat engine. Global maps of climatological variation in the maximum power estimates of (*B*) surface temperatures and (*C*) turbulent fluxes. Comparison of estimated (maximum power) and observed (CERES) (*D*) surface temperatures and (*E*) turbulent fluxes (ERA-5).

To address this challenge, we provide an alternative approach by viewing turbulent land surface exchange in the framework of a thermodynamic system. The key idea is to explicitly consider the second law of thermodynamics in addition to surface energy balance (25–28). The second law sets the direction of energy conversions and limits the total power generated out of a heating difference by requiring an overall increase in entropy. This outcome is then reflected in the well-established Carnot limit of heat engines. We apply this framework to surface–atmosphere exchange by describing the vertical convective transport in the land–atmosphere system as the consequence of a heat engine being driven by the heating difference between the warmer surface and the cooler atmosphere (Fig. 1*A*). Over land, the surface–atmosphere exchange is primarily shaped by solar radiative heating and the buoyancy that this generates. This is quite different to ocean surfaces, where solar radiation penetrates the surface ocean so that diurnal variations are buffered (29). The atmosphere performs work to maintain the exchange of turbulent fluxes and sustain vertical motion. This upward flux involves the transport of both heat and moisture. The rate of moisture input by evaporation is further limited by saturation at the surface, resulting in the concept of equilibrium partitioning of energy. The main difference between heat engines and the atmosphere is that the former is in contact with two heat reservoirs, meaning that the heat flux between those does not affect their temperature difference. This is essentially different in the case of the atmosphere, as on the one hand, the higher temperature difference between the two reservoirs of the heat engine will increase the turbulent flux exchange. On the other hand, increased turbulent fluxes will reduce the driving temperature difference through a continuous transport of heat away from the surface. This flux-gradient feedback and the related trade-off results in an optimal limit that maximizes the convective power generated by the atmosphere and is referred to as the maximum power limit. This framework has already been successfully tested against observations (27, 28). Here, we evaluate this approach at a global scale using satellite-derived observations of radiative forcings from National Aeronautics and Space Administration - Clouds and the Earth’s Radiant Energy System (NASA-CERES) (30, 31) and show that the estimates of turbulent fluxes and resulting surface temperatures at maximum power match corresponding observations at the continental and seasonal scale very well. This corroborates that the total magnitude of turbulent flux is thermodynamically constrained and depends predominantly on the local radiative conditions and the ability of the atmosphere to perform work. This then implies that the predominant effect of hydrologic cycling on land surface temperatures should be through radiative effects.

We tested this implication by evaluating the variation of land surface temperatures between dry and humid regions. These regions differ in their soil water availability near the surface which influences local evaporation and, in the atmosphere, where water affects clouds and thereby radiative fluxes. We then inferred evaporation from our approach, tested it with global observational datasets, and evaluated its role in surface energy balance partitioning across regions with different aridity. The impacts of clouds on surface temperatures were quantified across this gradient by using the “all-sky” and “clear-sky” radiative fluxes from the NASA-CERES dataset (30, 31) as forcing to our thermodynamically constrained energy balance model. With this approach, we are then able to discriminate the role of clouds vs. evaporation in shaping surface temperatures across regions with contrasting aridity.

## Results and Discussion

### Evaluation of Maximum Power Limit with Observations.

We start with the evaluation of our approach to estimate surface temperatures and surface energy balance partitioning over land from maximum power with observations at the continental scale. Turbulent fluxes and surface temperatures were calculated by maximizing the power of convective exchange associated with a heat engine operating between the surface and the atmosphere. The estimated surface temperatures and optimized turbulent fluxes using the maximum power limit are compared to those inferred from the NASA-CERES and ERA-5 dataset, respectively, in Fig. 1. For this evaluation, surface temperatures from NASA-CERES were derived from the upwelling longwave radiation, and the turbulent flux data were derived from ERA-5 as the sum of the sensible and latent heat flux. We find a strong agreement with r^2^ > 0.9 for both mean surface temperatures and turbulent fluxes (Fig. 1 *D* and *E*). Similar results were found when the optimized turbulent fluxes were compared with the FLUXCOM (32) and FLUXNET-2015 (Pastorello et al., 2020) datasets (*SI Appendix*, Fig. S1). Estimated surface temperatures from maximum power and those derived from NASA-CERES were also compared with the ERA-5 land surface temperature data (*SI Appendix*, Fig. S2). The consistency of results was also checked for the seasonal amplitudes (*SI Appendix*, Fig. S3). Monthly rmse remains less than 4 K throughout the year (*SI Appendix*, Fig. S4*A*). We have not considered the effect of ground heat flux as its magnitude is typically much lower than turbulent fluxes, which might be reflected in the rmse (*SI Appendix*, Fig. S4*B*). While some distinct biases can be seen (*SI Appendix*, Fig. S4), our approach captures the broad climatological variation remarkably well. What this implies is that the atmosphere appears to work at an optimal limit to exchange turbulent fluxes that maximize the convective power. Thermodynamics thus imposes a major constraint on turbulent flux exchange, which in turn is primarily determined by the radiative forcing.

Next, we performed the partitioning of the optimized turbulent fluxes into sensible and latent heat to identify the role of evaporation for surface temperatures. When water is sufficiently available, the partitioning was done using the thermodynamic equilibrium partitioning (*SI Appendix*, *Text A2*). This partitioning represents the limit for evaporation at the surface as it assumes that the heat added to the atmosphere is partitioned according to the thermodynamic equilibrium between heating and moistening of air. These proportions are described by fractions that depend on temperature and are very well established in micrometeorological approaches to estimate evaporation (e.g., refs. 33 and also ref. 25). If water is limited, then we used the ratio of actual to potential evaporation from the GLEAM evaporation dataset (34), with the ratio referred to as the water limitation factor (f_W_). The water limitation factor is essential to capture the reduced evaporation over dry surfaces which cannot be captured by the equilibrium partitioning. However, it does not affect the maximum power trade-off or the magnitude of optimized turbulent fluxes. The resulting estimates for the sensible and latent heat flux (*SI Appendix*, Fig. S5) compare well (r^2^ > 0.7) with the FLUXCOM dataset (32). This consistency with observations shows that while the antecedent hydrologic conditions are clearly important to the energy partitioning into sensible and latent heat, the first-order control on the total turbulent flux exchange is mainly determined by radiative and thermodynamic constraints.

### Role of Hydrologic and Radiative Constraints Shaping LSTs.

**Fig. 2.**
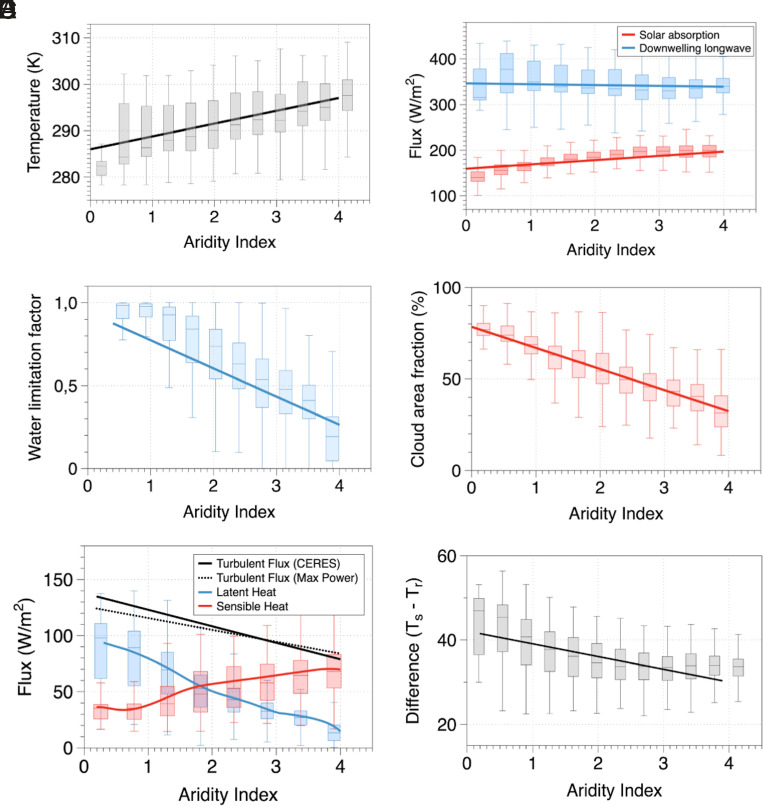
Variations along the AI of (*A*) land surface temperatures, (*B*) surface absorption of solar (red) and downwelling longwave radiation (blue), (*C*) water limitation factor defined as the ratio of actual to potential evaporation, (*D*) cloud area fraction (%), (*E*) turbulent fluxes estimated at maximum power (black dotted line), turbulent fluxes derived from CERES observations (black solid line), partitioned fluxes into sensible (red) and latent heat (blue), and (*F*) difference between the source and sink temperature of the conceptualized heat engine. Note that the surface temperature is representative of the source, while the radiative temperature of the atmosphere is representative of the sink temperature.

To understand the effects of evaporation vs. clouds on the temperature at the climatological scale, we next look at the variations in the surface energy balance with increasing aridity. For this, we used the Aridity Index (AI), which is defined as the ratio of the potential evaporative water demand to mean annual precipitation water supply (R_net_/LP) (35), the former being estimated by its energy limit net radiation, divided by the specific heat of vaporization L. Lower AI values indicate humid regions, while higher AI values are associated with dry regions. As one may expect, we find an increase in land surface temperatures with aridity, indicating that drier regions are generally warmer than humid regions (Fig. 2*A*).

While this may seem intuitively clear, the cause for this trend is not so straightforward. On the one hand, dry regions are water limited and evaporate less. This trend can be clearly seen by the decrease in water limitation and evaporation with aridity (blue line in Fig. 2 *C* and *E*). On the other hand, arid regions have less clouds, so the absorbed solar radiation increases with aridity (red line in Fig. 2 *B* and *D*). Although arid regions also have a higher surface albedo, we show that changes in absorbed solar radiation with aridity are largely due to decrease in cloud cover (*SI Appendix*, Fig. S6). Downwelling longwave radiation is largely insensitive to aridity (Fig. 2*B*), which can be understood in terms of a semiempirical formulation for this radiative flux by ref. 36 (*SI Appendix*, *Text A3* and Fig. S7). This leads us to the question of whether the warmer temperatures in arid regions are primarily caused by reduced evaporation or less clouds.

**Fig. 3.**
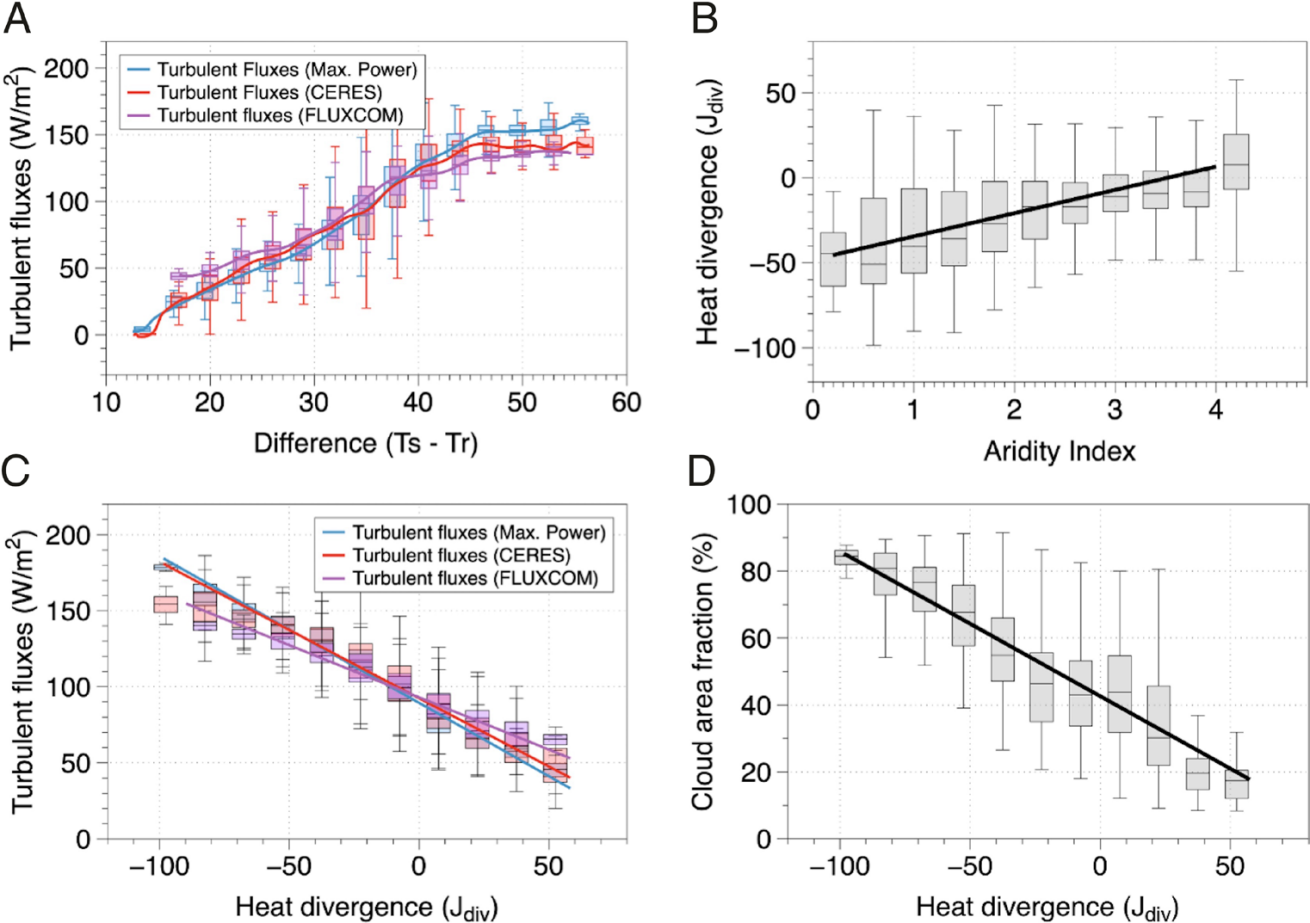
(*A*) Variation of turbulent fluxes observations from CERES (red), FLUXCOM (purple), and estimated fluxes at maximum power (blue) with the temperature difference between the surface and the atmosphere. Surface and atmospheric temperatures were derived from upwelling and outgoing longwave flux at the surface and atmosphere, respectively, from CERES. (*B*) Variation of heat divergence (calculated as the difference between outgoing longwave and incoming shortwave radiation at the top of the atmosphere) with AI, (*C*) variation of turbulent fluxes with heat divergence, and (*D*) variation of cloud area fraction (%) with heat divergence.

To address the role of evaporation, we first note that the total turbulent heat fluxes decrease with aridity (Fig. 2*E*). This trend in the fluxes inferred from the CERES dataset (and consistent in FLUXCOM and ERA-5, *SI Appendix*, Fig. S8) is very well captured by the maximum power limit, so it can be explained by the change in radiative forcings for the heat engine with an increase in aridity. We attribute this decrease in turbulent fluxes to the decrease in the driving temperature difference of the heat engine, T_s_ − T_r_ (Fig. 2*F*). This lowers its efficiency and results in a different outcome of the maximum power limit. This decrease in energy efficiency originates from the difference in the radiative imbalance at the top of the atmosphere that is shaped by the large-scale atmospheric circulation, particularly the Hadley circulation. Tropical humid regions are shaped by strong precipitation, deep convection, and a low flux of outgoing longwave radiation at the top of the atmosphere, representing the rising branch of the Hadley cell. This results in a low radiative temperature and a large temperature difference between the surface and the atmosphere, resulting in greater efficiency of the heat engine. Subtropical arid regions are predominantly found in areas where the Hadley cell descends and brings heat. This heat is eventually lost by increased fluxes of outgoing longwave radiation. This leads to higher radiative temperatures and a reduced efficiency of the heat engine. The maximum power limit results from the trade-off between greater heat flux and lower efficiency. When the temperature difference is reduced due to the different radiative conditions in arid regions, then this trade-off is weaker, resulting in lower optimum heat fluxes. This effect is seen in a clear variation of turbulent fluxes with this temperature difference in observations and the maximum power limit (Fig. 3*A*).

To make this point further clear, we use divergence of atmospheric heat transport (J_div_) which we define as the difference between outgoing longwave and incoming shortwave radiation at the top of the atmosphere (R_l, toa_ – R_s, toa_). Positive and negative J_div_ implies a net import and export of heat respectively from different regions. When plotted against aridity, we see an increase in J_div_ as we move toward more dry regions (Fig. 3*B*). This is consistent with our reasoning of large-scale circulation patterns like Hadley circulation which transport heat in the drier subtropics. Next, we plot turbulent fluxes from CERES and FLUXCOM against heat divergence and found a very clear relationship indicating less turbulent fluxes with more J_div_ (Fig. 3*C*). Maximum power estimates reproduce this relationship very well and add a physical explanation behind such an effect through the weakening of the heat engine.

This explains why our thermodynamically constrained surface energy balance model predicts turbulent fluxes very well across the globe without accounting for surface information on water availability. What this implies is that the decrease in mean turbulent flux with aridity does not relate to reduced evaporation but rather to the prevailing radiative conditions at the surface and the top of the atmosphere. This interpretation has important implications. It explains why the sensible heat flux compensates for the decrease in latent heat flux with greater aridity (Fig. 2*E*), resulting in greater buoyancy production in arid regions. This compensating effect is also seen in observations and ERA-5 (*SI Appendix*, Fig. S8). Hence, it would seem that reduced evaporation is not the main cause for the warmer mean surface temperatures in more arid regions.

### Quantifying the Role of Clouds.

The subsidence over dry regions associated with increased divergence of heat transport (J_div_) also results in less clouds (Fig. 3*D*). Hence, with increasing aridity, it is not just the water availability that is reduced, but cloud cover decreases as well (Fig. 2*D*). In fact, the water limitation factor strongly correlates with cloud cover, with three distinct regimes labeled R1, R2, and R3 shown in *SI Appendix*, Fig. S9. The second regime (R2) relates closely to regions that were previously identified to have soil moisture control on the surface energy partitioning (8, 37 and *SI Appendix*, Fig. S9). It is important to note that the presence of clouds is not merely a result of local recycling and evaporation but of large-scale moisture advection and circulation patterns. What the correlation of water limitation with cloud cover then implies is that these regions are also shaped by strong variations in cloud cover and thus differences in radiative forcing.

**Fig. 4.**
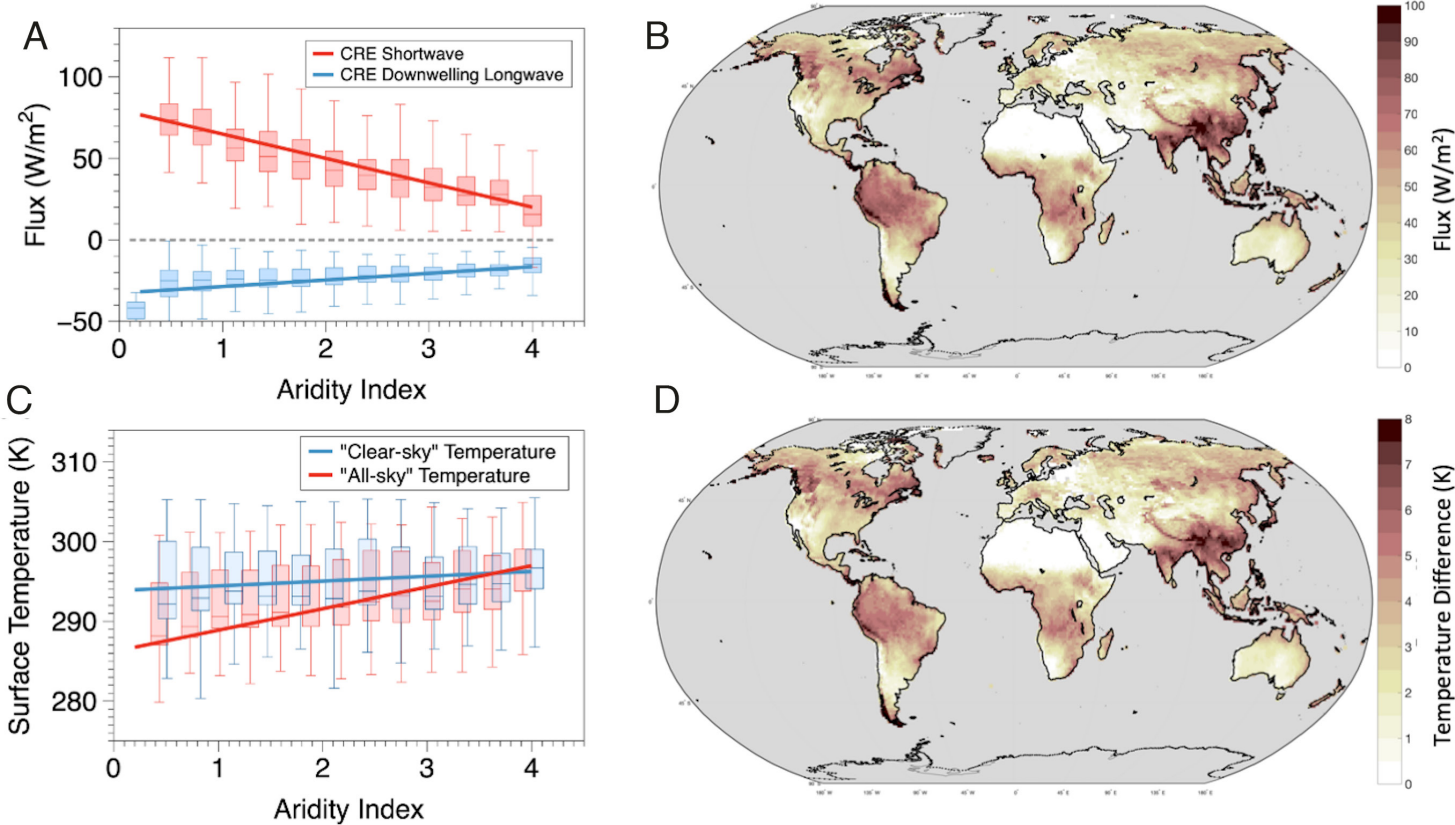
Variation of (*A*) CRE radiative fluxes defined as the difference between clear-sky and all-sky conditions for shortwave (red) and downwelling longwave (blue) radiations along the AI, (*B*) global map of the climatological variation in total CRE, (*C*) variation of estimated clear-sky temperatures (temperature without the clouds) and all-sky temperatures (temperature with observed conditions) along the AI, and (*D*) global map of cloud radiative cooling of surface temperatures calculated as the temperature difference between clear-sky and all-sky conditions.

We quantified these effects by using satellite observations from NASA-CERES (31) and looking at differences between clear-sky and all-sky radiative conditions (Cloud radiative effects, CRE) as a function of aridity. We show that the clouds reduce the incoming shortwave at the surface (red line in Fig. 4*A*) by more than 100 W/m^2^ over humid regions, while the changes in downwelling longwave radiative flux (blue line) remain relatively lower. The strength of these effects reduces as we move toward the drier regions. The global map of total CRE (shortwave + longwave) at the surface (Fig. 4*B*) shows a systematic decrease in the incoming energy over humid regions, while these changes tend to disappear over arid regions. This then leads us to the question of how these changes in radiative conditions associated with clouds translate to changes in surface temperature.

To quantify this, we used clear-sky and all-sky radiative fluxes as forcing to our thermodynamically constrained surface energy balance model and estimated clear-sky and all-sky temperatures. Clear-sky temperatures are representative temperatures at the surface considering no cloud cover, while the all-sky temperatures are representative of observed conditions and have already been shown to be consistent with observations (Fig. 1*D*). We then used the difference between clear-sky and all-sky temperatures as a metric to quantify the cloud cooling effects and present its geographical map in Fig. 4*D*. We find that the CRE on surface temperatures is stronger in the humid tropics where clouds cool the surface by as much as 7 K, while these effects disappear over arid regions. The strongest cloud radiative cooling can be seen in Southeast Asia, the Indian monsoon region, Northeast America, Central Africa, and the Amazon. When plotted against the AI, clear-sky temperatures (blue line in Fig. 4*C*) remain insensitive to changes in aridity, while all-sky temperatures (red line) show an increase, consistent with observations (Figs. 3*A* and 4*C*). This indicates that the radiative effects induced by clouds can explain the increase of surface temperatures with aridity and seem to be the predominant reason which makes arid and humid regions warmer and cooler, respectively.

These results then relate back to our interpretation of radiation as the predominant driver behind the climatological variations in land surface temperatures. Our findings on how radiation affects surface temperatures can be summarized by the following mechanisms. First, the local radiative conditions together with the thermodynamic limit constrain the vertical exchange of turbulent fluxes of sensible and latent heat. This is reflected in the high level of consistency between the maximum power estimates and observations across the globe. This further explains the increase in sensible heat over dry regions which compensates for the reduced evaporation due to lack of water. More sensible heat then results in increased buoyancy which is also consistent with the higher growth of the atmospheric boundary layer observed during drier conditions (38).

Second, the radiative imbalance between the incoming and outgoing radiation at the top of the atmosphere captures the effects of heat transport and large-scale atmospheric circulation. Heat transport into a region increases the radiative temperature of the atmosphere as more heat is then emitted back to space. This in turn suppresses the driving temperature difference and reduces the efficiency of the atmospheric heat engine. This can be seen in Figs. 2*F* and 3*B*. The reduced efficiency in turn affects the ability of the surface to maintain the vertical exchange of both heat and mass and results in the reduction of turbulent fluxes with aridity (Fig. 2*E* and *SI Appendix*, Fig. S8). This is not in contradiction with increased buoyancy during drier conditions because of reduction in mass exchange as a result of less evaporation.

Last, the reduced abundance of clouds toward more arid regions changes the radiative conditions and affects the available energy at the surface. We showed that these CREs modulate the variations in surface temperatures across the dry and humid regions at the climatological scale. The availability of water at the surface seems to not have a large effect on the climatological variation in land surface temperature which can be attributed to the more dominant controls imposed by radiation and thermodynamics. At present, we do not make any assumption about clouds being a result of moisture advection or local recycling as it will not affect our results at a climatological scale. Our results imply that the role of evaporation on continental land surface temperatures is not determined by evaporative cooling at the surface but by the ability of evaporation to affect the local cloud cover. However, at shorter timescales, soil water limitations may amplify the local feedback by adding more sensible heat into the atmosphere which can lead to enhanced heating that typically sustains droughts and heatwaves (14; Zhou et al., 2019).

Local effects (such as different evaporation from forested or deforested land or increased evaporation by abundance of wetlands) can impact temperature and turbulent fluxes through different mechanisms by changing surface albedo (10), aerodynamic conductance (11), surface water availability conditions (39), and by feeding back to changes in cloud cover (40). By using observations of absorbed radiative forcings and cloud area fraction as inputs, our model indirectly considers albedo and cloud effects that arise from vegetation changes. Other local effects may primarily explain variability around the mean response, but further analysis of land cover change is beyond the scope of this study.

It is important to note that our objective here is not to explain all the variability in land surface temperatures and turbulent fluxes but rather to determine the predominant constraints that shape most of the climatological variations. Our idealized heat engine framework assumes a black atmosphere such that all radiation emitted from the surface is absorbed. This ignores the effect of the atmospheric window which may result in biases (41). The present approach does not take into account the temperature inversion conditions predominant at high latitudes. While these issues can be addressed by performing a detailed parameterization of radiative transfer and explicitly considering the boundary layer dynamics, the strong agreement of our estimates with observations is remarkable and shows that we capture the predominant effects very well.

Our simple physics-based approach takes a step back from model complexity and focuses on determining the first-order controls that shape climate over land. While the need for having a hierarchy of models of varying complexity to better understand the climate system has already been emphasized (42), we aim to fill this gap with our approach that solely relies on physical principles. Although our description of land surface exchange is quite different compared to how these fluxes are described in Earth system models, it provides additional value about the relevant physical constraint primarily arising from radiation and thermodynamics that shapes these estimates. We show that the atmosphere works at its thermodynamic limit to maximize the exchange of turbulent fluxes. Our interpretation is also consistent with previous research that has applied thermodynamic principles to atmospheric dynamics and has shown that atmospheric processes organize themselves to an optimum state (43–46).

## Conclusion

In this study, we show that radiation and the thermodynamic limit of maximum power set the main controls on the climatological and seasonal variations in land surface temperatures and predict them very well across continents. We used a thermodynamic theory that characterizes the turbulent flux exchange of sensible and latent heat as a result of work performed by an idealized heat engine operating between the warmer surface and cooler atmosphere. We show that the atmosphere maximizes the convective power to sustain vertical exchange for given radiative conditions, thus imposing a major constraint on turbulent fluxes. This implies that while the availability of water over land strongly affects the partitioning of available energy into sensible and latent heat, it does not alter the total amount of turbulent fluxes, which is primarily constrained by radiative conditions at the surface, top of the atmosphere, and thermodynamics. The main effect of hydrologic cycling on surface temperatures is then modulated mostly by clouds that alter the mean radiative environment across dry and humid regions.

By invoking the thermodynamic limit of maximum power, our approach substantially simplifies the inherent complexities in land surface exchange. It highlights the importance of physical constraints arising from radiation and thermodynamics in mediating the conditions of the land–atmosphere system, including its many interactions. It can further help to increase our understanding about the response of land–atmosphere fluxes to changes in land cover, their interactions with vegetation, and their sensitivity to global warming.

## Methods and Datasets

### Thermodynamically Constrained Surface Energy Balance Model.

Solar radiation continuously heats the Earth’s surface making it warmer. This energy is then released back at a much colder temperature from the top of the atmosphere. This temperature difference creates a thermal disequilibrium that is depleted by the exchange of turbulent fluxes between the surface and atmosphere. We formulated a surface energy balance model that conceptualizes the turbulent flux exchange as an outcome of an idealized heat engine (Fig. 1*A*) operated between the hot Earth’s surface (as a source) and the cold atmosphere (as a sink). We used the radiative fluxes of solar absorption and downwelling longwave radiation as the forcing to our heat engine model. The source and sink temperature were determined by the upwelling longwave radiation at the surface and the outgoing longwave radiation from the top of the atmosphere respectively. Turbulent fluxes were then predicted by maximizing the power that the heat engine can generate (*SI Appendix*, *Text A1*). These estimates were then evaluated against the observational-based datasets. Their results were used to interpret our understanding of the variations in land surface temperatures. This approach has been described in ref. 27; (25), and further details can be found in *SI Appendix*, *Text A1*.

### Datasets for Model Forcings.

We used all-sky and clear-sky radiative fluxes at the surface and top of the atmosphere and from NASA-CERES EBAF 4.1 dataset (DOI: 10.5067/TERRA-AQUA/CERES/EBAF_L3B.004.1) and also tested it with ERA-5 dataset (47, DOI: 10.24381/cds.f17050d7). Details on all the variables with their notations are mentioned in *SI Appendix*, Table S1. Data on cloud area fraction and CRE were also derived from NASA-CERES (EBAF ed 4.1). To calculate the water limitation factor as the ratio of actual to potential evaporation (E_act_/E_pot_), actual and potential evaporation data were used from the GLEAM V3.6b dataset (http://www.gleam.eu). To calculate the AI as the ratio of mean annual net radiation to the energy equivalent of mean annual precipitation (R_net_/LP), rainfall data from GPCP V1.3 (http://doi.org/10.7289/V5RX998Z) were used, while the net radiation was derived from CERES EBAF 4.1.

### Datasets for Model Evaluation.

Estimated turbulent fluxes were evaluated against data from FLUXCOM, FLUXNET, ERA-5, and CERES EBAF 4.1. Monthly sensible and latent heat data at (0.5° × 0.5°) grid resolution were obtained from the FLUXCOM dataset (32). To validate the results against the flux tower observations, the FLUXNET-2015 dataset was used (48). After the post-data processing and ensuring the continuous availability of all the variables, 109 sites were used for validation. Details on each site are mentioned in *SI Appendix*, Table S2. To validate results against CERES, monthly global net radiation was used as a proxy for turbulent fluxes from CERES EBAF ed4.1 dataset available at (1° × 1°) grid resolution (30, 31).

## Supplementary Material

Appendix 01 (PDF)

## Data Availability

All the datasets used in the present study and described in (Methods and Datasets section and Table T1 in SI) are publicly available. NASA-CERES data is accessible from https://asdc.larc.nasa.gov/project/CERES/CERES_EBAF_Edition4.1 (49). ERA-5 data is accessible from https://cds.climate.copernicus.eu/cdsapp#!/dataset/10.24381/cds.f17050d7?tab=overview (47). GLEAM dataset is accessible from https://www.gleam.eu/ (34). FLUXCOM data is accessible from https://www.fluxcom.org/EF-Download/ (32). GPCP rainfall data is accessible from https://www.ncei.noaa.gov/access/metadata/landing-page/bin/iso?id=gov.noaa.ncdc:C00999 (50). FLUXNET-2015 dataset is accessible from https://fluxnet.org/data/fluxnet2015-dataset/ (48). The codes to implement the maximum power approach are accessible from https://doi.org/10.17617/3.HNDICH (51).
