# Supplementary material for: Radiative controls by clouds and thermodynamics shape surface temperatures and turbulent fluxes over land
Source: Proc Natl Acad Sci U S A. 2023 Jul 10;120(29):e2220400120. doi: 10.1073/pnas.2220400120 (PMC10629566; doi:10.1073/pnas.2220400120)
Supplement: Supplementary file 1 — Appendix 01 (PDF) [file pnas.2220400120.sapp.pdf]

PNAS

Supplementary Information for

Radiative controls by clouds and thermodynamics shape surface temperatures and turbulent fluxes over land

Sarosh Alam Ghausi[1,2], Yinglin Tian[3], Erwin Zehe[4] and Axel Kleidon[1]
[1] Biospheric Theory and Modelling Group, Max Planck Institute for Biogeochemistry, Jena 07745, Germany.
[2] International Max Planck Research School for Global Biogeochemical Cycles (IMPRS – gBGC), Jena 07745, Germany.
[3] Department of Hydraulic Engineering, Tsinghua University, 100084 Beijing, China
[4] Institute of Water Resources and River Basin Management, Karlsruhe Institute of Technology – KIT, Karlsruhe, Germany

Corresponding author: Sarosh Alam Ghausi **<sghausi@bgc-jena.mpg.de>**

This PDF file includes:

Supplementary text: A1 to A3
Figures S1 to S9
Table T1 to T2
SI References

Text A1: Thermodynamically constrained surface energy balance model:

A1.1: Model Conceptualization

The Earth's surface is continuously heated by incoming solar radiation, which makes it warmer. This energy is then released back by the earth into the atmosphere. However, this emission takes place at top of the atmosphere at a much lower temperature than Earth's surface. This temperature difference between the surface and the atmosphere drives the exchange of heat and mass (turbulent fluxes) as the surface-atmosphere system tries to achieve a state of thermal equilibrium.

We conceptualize this transfer as a result of a heat engine (Figure 1) operating between the warmer earth's surface and the cooler atmosphere. We then explicitly considered the second law of thermodynamics to quantify and constrain this transfer following the approach shown in (Kleidon & Renner, 2013; Dhara et al., 2016; Kleidon et al., 2018) and briefly described below.

A1.2 Deriving the thermodynamic limit

We start by applying the first law of thermodynamics to the conceptualized atmospheric heat engine, which is given by equation 1.

$$\frac{dU}{dt} = J_{in} - J_{out} + D - G \qquad (1)$$

Where J_{in} represents the heat added into the system from the hot source (surface) through the exchange of turbulent fluxes, J_{out} represents the total heat exported out of the heat engine at the cold sink (atmosphere). G denotes the total power generated by the engine to sustain vertical mixing while D denotes the energy associated with the frictional dissipative heating. We assumed a steady state where the total power generated balances the frictional dissipation (G = D). dU/dt denotes the seasonal heat storage and heat transport changes within the system (Kleidon et al., 2018) and is expressed as in equation 2.

$$\frac{dU}{dt} = R_s - R_{l,toa} \qquad (2)$$

R_s and $R_{l,toa}$ in equation 2 are the absorbed solar radiation and outgoing longwave radiation respectively. The second step is to write the entropy budget for the system (second law of thermodynamics). It includes the entropy added into the system by turbulent fluxes (J_{in}) at hot source temperature, entropy exported out by radiative cooling (J_{out}) at cold sink temperature, entropy associated with heat storage changes, and entropy generated by the frictional dissipation. We consider an idealized case where no entropy is produced from any other irreversible processes besides frictional dissipation. The change in entropy of the system is then given by equation 3.

$$\frac{1}{T_s}\frac{dU}{dt} = \frac{J_{in}}{T_s} - \frac{J_{out}}{T_r} + \frac{D}{T_s} \qquad (3)$$

The source and sink temperatures were defined as the temperature of the earth's surface (T_s) and the radiative temperature of the atmosphere (T_r) respectively. It is to note that dissipation D

primarily occurs in the lower atmosphere where mixing happens. The entropy associated with this term should ideally correspond to the potential temperature of lower atmosphere. As surface is closer to the lower atmosphere, we make an assumption to use surface temperature instead. Similar assumption has been made in previous studies (Kleidon & Renner 2018; Conte et al., 2019). The surface and radiative temperatures were derived from the upwelling longwave radiation ($R_{l,up}$) and outgoing longwave radiation ($R_{l,toa}$) respectively from equations 4 and 5.

$$T_s = \left(\frac{R_{l,up}}{\sigma}\right)^{\frac{1}{4}} \quad (4)$$

$$T_r = \left(\frac{R_{l,toa}}{\sigma}\right)^{\frac{1}{4}} \quad (5)$$

By replacing J_{out} from equation 1 and combining it with equation 3, we then get the expression for power (G) generated by the atmosphere which is given by equation 6.

$$G = \left(J_{in} - \frac{dU}{dt}\right)\left(\frac{T_s - T_r}{T_r}\right) \quad (6)$$

The resulting expression in absence of atmospheric heat storage change is very similar to the widely known Carnot limit and have been referred to as the thermodynamic limit for cold heat engine (Kleidon et al., 2018). The equation 6 can be rewritten in terms of turbulent flux (J_{in}) using the surface energy balance and equation (4) as in equation 7.

$$G = \left(J_{in} - \frac{dU}{dt}\right)\left(\frac{\left(\frac{R_s + R_{ld} - J_{in}}{\sigma}\right)^{\frac{1}{4}}}{T_r} - 1\right) \quad (7)$$

A1.3: Maximum power trade-off

Based on equation 3, the convective power generated by the atmosphere to sustain vertical motion depends on the turbulent flux exchange (J_{in}), heat storage changes (dU/dt), and the difference between the surface and radiative temperature (T_s – T_r). However, this temperature difference is not a fixed property of the system as there exists a covariation between the terms of turbulent flux exchange (J_{in}) and the temperature difference (T_s – T_r). On one hand, a higher temperature difference between the surface and atmosphere will increase the turbulent flux exchange. On the other, increased turbulent fluxes will imply more evaporative cooling at the surface and condensational heating in the atmosphere which will deplete the driving temperature difference (T_s – T_r). This trade-off leads to a maximum in power for an optimum turbulent flux (J_{opt}) and is also reflected in equation 6. This is referred to as the maximum power limit. This optimum flux was calculated at the maximum power limit by numerically solving equation 8.

$$\frac{dG}{dJ} = 0 \quad (8)$$

J_{in} was varied within the limits of heat engine from J_{in} = 0 (no surface cooling by convection) to J_{in} = J_{max} = R_s + R_{ld} - σT_r^4. J_{max} represents a case, where Ts- Tr = 0 and there is no thermal disequilibrium within the heat engine anymore to drive J and J reaches its theoretical maximum value. Also, it is to note that dU/dt is not zero in the solution of equation 8, It results in an offset and thereby does not affect the maximum power trade-off but affects the magnitude of optimized turbulent flux (J_{opt}).

A1.4 Estimation of surface Temperatures

The surface temperatures at maximum power were then calculated using the surface energy balance together with the optimised turbulent fluxes using equation 9.

$$T_{\max power} = \left(\frac{R_S + R_{ld} - J_{opt}}{\sigma}\right)^{\frac{1}{4}} \qquad (9)$$

Here R_s is the absorbed solar radiation, R_{ld} is the downward longwave radiation, J_{opt} is the optimal turbulent flux that maximizes the convective power in equation 6 and σ is the Stefan – Boltzmann constant with the value of 5.67 * 10^{-8} $Wm^{-2}K^{-4}$.

A1.5 Removing the cloud radiative effects from surface temperatures

To remove the cloud radiative effects from surface temperatures, we used the "clear-sky" fluxes from the NASA-CERES dataset as forcing to our thermodynamically constrained formulation of surface energy balance. "Clear-sky" fluxes from NASA – CERES are diagnosed by removing the clouds from the radiative transfer. More details can be found here (Loeb et al., 2018; Kato et al., 2018).

We first numerically calculated the maximum convective power generated from the clear-sky fluxes by solving equation 8 and then use it to estimate the "Clear-sky" temperatures using equation 10.

$$T_{Clear\ sky} = \left(\frac{R_{s,Clear\ sky} + R_{ld,Clear\ sky} - J_{opt}}{\sigma}\right)^{\frac{1}{4}} \qquad (10)$$

Text A2: Surface Energy partitioning

To partition the optimized turbulent fluxes estimated from the maximum power limit into sensible and latent heat, we used the equilibrium energy partitioning approach (Slayter & McIlroy, 1961; Priestley & Taylor, 1972) and also described in Kleidon & Renner (2013). This framework however assumes a saturated surface with no water limitation. To apply it at a global scale, we account for water limitation by introducing a limitation factor (f_w). This factor was calculated as the ratio of actual to potential evaporation using GLEAM V3.6b data (Martens et al., 2017). Latent heat and sensible heat were then calculated from equations 11 and 12.

$$LE = f_w \frac{s}{s+\gamma} J_{opt} \qquad (11)$$

$$H = J_{opt} - LE \qquad (12)$$

Where s and γ are the slope of the saturation vapor pressure curve and Psychometric constant respectively. J_{opt} is the optimized turbulent flux estimated from maximum power limit.

Text A3: Decomposition of Downwelling longwave radiation

Downwelling longwave radiation largely depends on how hot and black the atmosphere is. On one hand, a hotter atmosphere will emit more radiation back to earth as a result of higher radiative temperature. On the other hand, the increase in emissivity of the atmosphere will lead to enhanced absorption and re-emission of downward longwave radiation. The former is likely to increase with enhanced heat transport while the latter largely depends on the amount of water vapor and clouds in the atmospheric column. To decompose these two effects, we used the semi-empirical formulation of downwelling longwave radiation proposed by Brutsaert (1975) and Crawford & Duchon (1999). Downwelling radiation can then be described by the following equation:

$$R_{ld} = \varepsilon \sigma T_a^4 \qquad (13)$$

Where σ is the Steffan Boltsman constant with the value of 5.67 x 10^{-8} $Wm^{-2}K^{-4}$. T_a is the near-surface air temperature and ε is the emissivity of the atmosphere which is a function of cloud area fraction and vapor pressure as described in equation 14.

$$\varepsilon = \left(f_c + (1 - f_c) 0.55 e_o^{\frac{1}{7}} \right) \qquad (14)$$

Here, f_c is the cloud fraction (0 -1) which was derived using NASA-CERES EBAF ed4,1 dataset and e_o denotes the actual vapor pressure. We first compared the estimated downwelling longwave radiation calculated from equation 14 with the observations from NASA-CERES (Loeb et al., 2018; Kato et al., 2018). We find strong agreement over global land with the R^2 value of 0.97. The differential form of equation 13 was then used to decompose the downwelling longwave radiation as shown in equation 15.

$$\Delta R_{ld} = \sigma \overline{T_a}^4 \Delta\varepsilon + 4\sigma\bar{\varepsilon}\overline{T_a}^3 \Delta T_a \qquad (15)$$

The first term in equation 15 shows the variation in R_{ld} due to changes in the emissivity of the atmosphere (blue line in figure S6) while the second term shows the changes in R_{ld} due to changes in the atmospheric temperature (Red line in figure S6).

Figures: S1

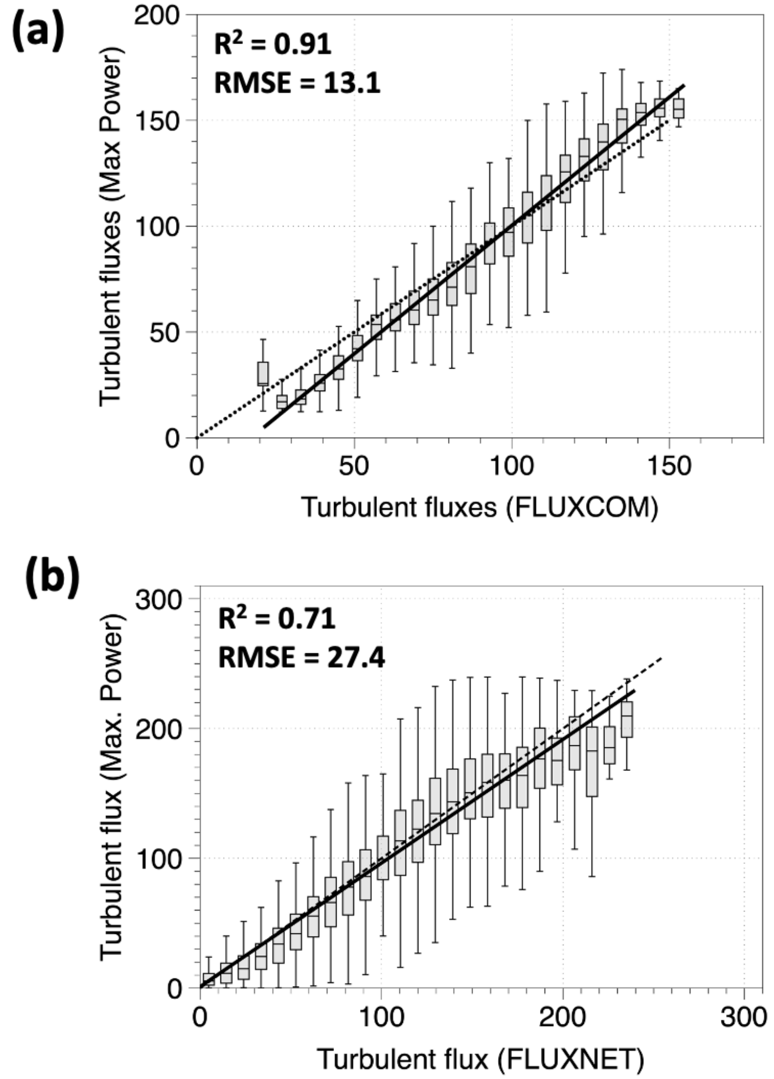

Figure S1: Same as figure (1e) but for (a) data from FLUXCOM (Jung et al., 2019) and (b) data from 109 FLUXNET sites (Pastoreallo et al., 2020).

Figure S2

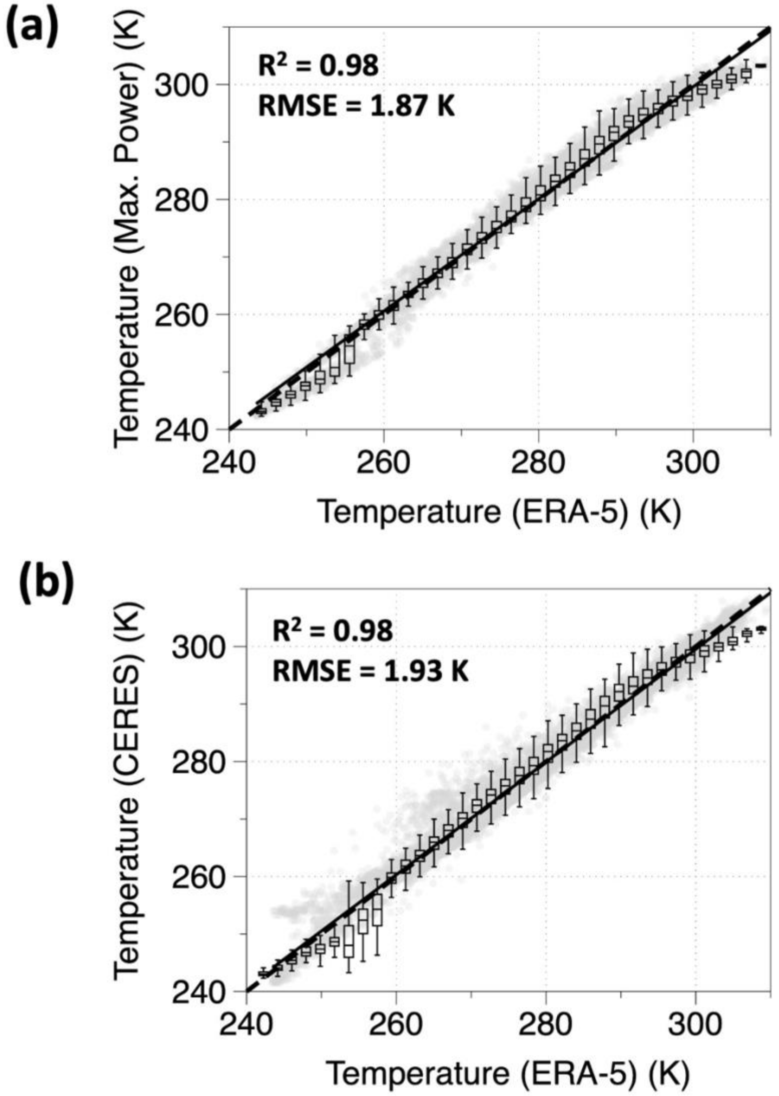

Figure S2: (a) same as figure 1d but for ERA-5 data (b) Comparison of mean surface temperatures over global land derived from CERES with ERA-5 surface temperature.

Figure S3

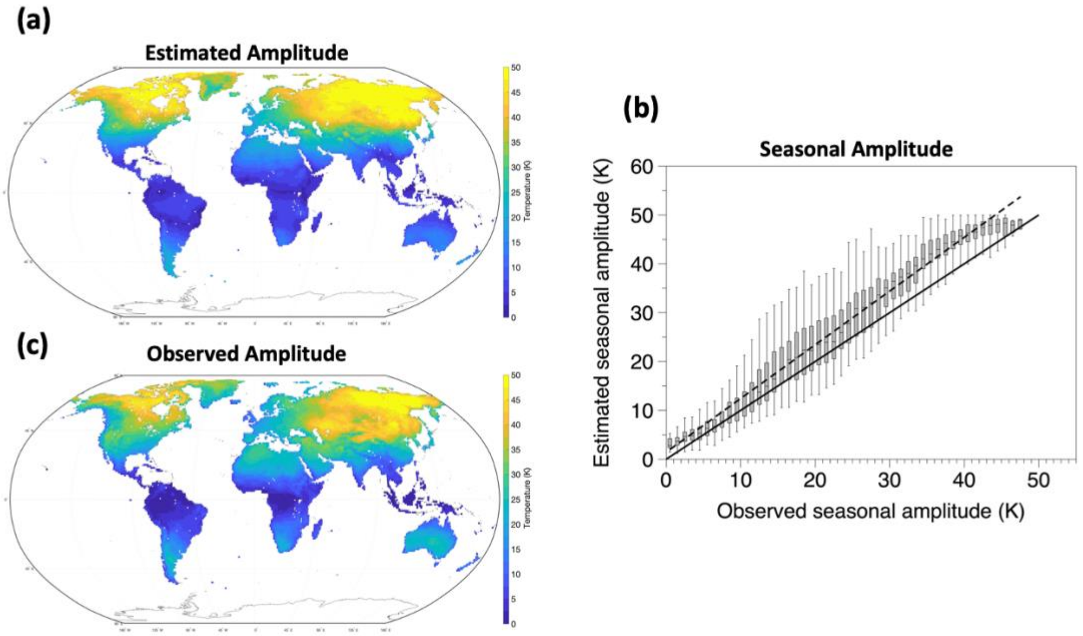

Figure S3: Global maps of the temperature amplitude (calculated as the difference between the maximum and minimum monthly temperatures) for estimated max power temperatures (a) and surface temperatures from CERES (c) along with their comparison (b) across the global land.

Figure S4

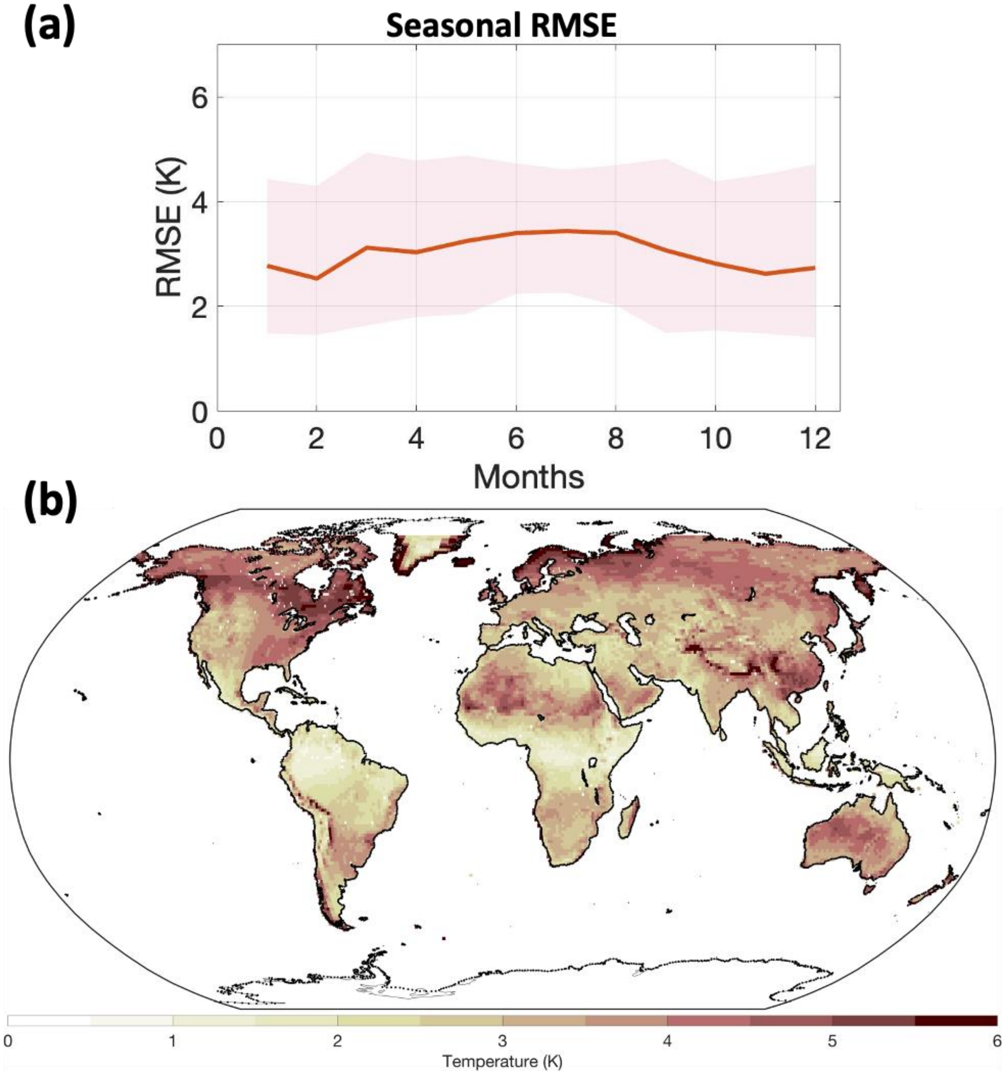

Figure S4: (a) Annual variation of monthly root mean squared error (RMSE) between estimated and observed surface temperatures averaged over all the grid points on global land. (b) global map of mean monthly RMSE at each grid point.

Figure S5

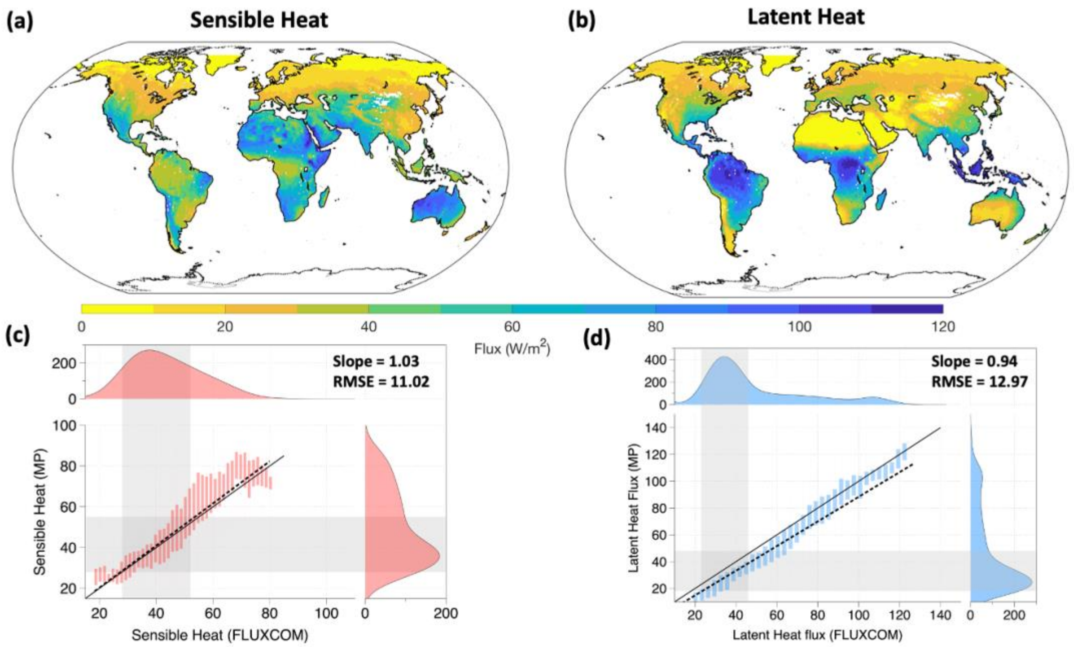

Figure S5: Global maps of climatological variation of partitioned optimal turbulent fluxes into Sensible heat (a) and Latent heat (b). Comparison of estimated fluxes with FLUXCOM for Sensible heat (c) and Latent heat (d).

Figure S6

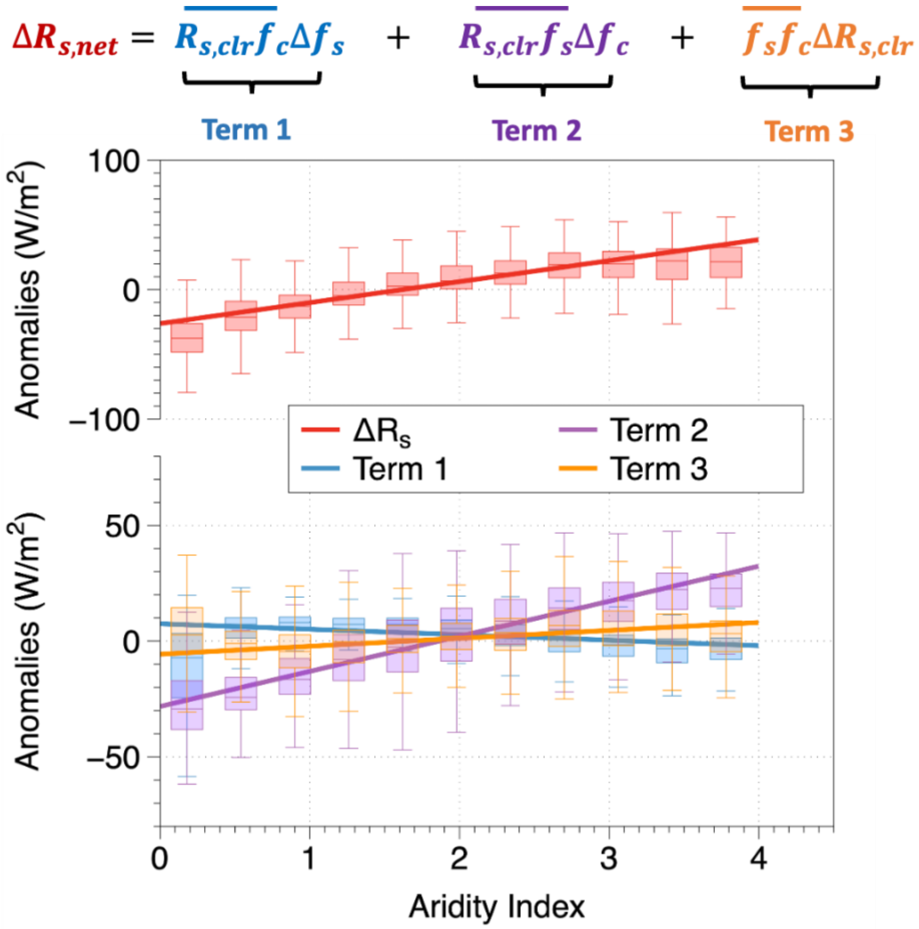

Figure S6: Variation of absorbed solar radiation (red) decomposed into term1 (blue), term2 (purple) and term3 (orange) with aridity index.

Figure S7

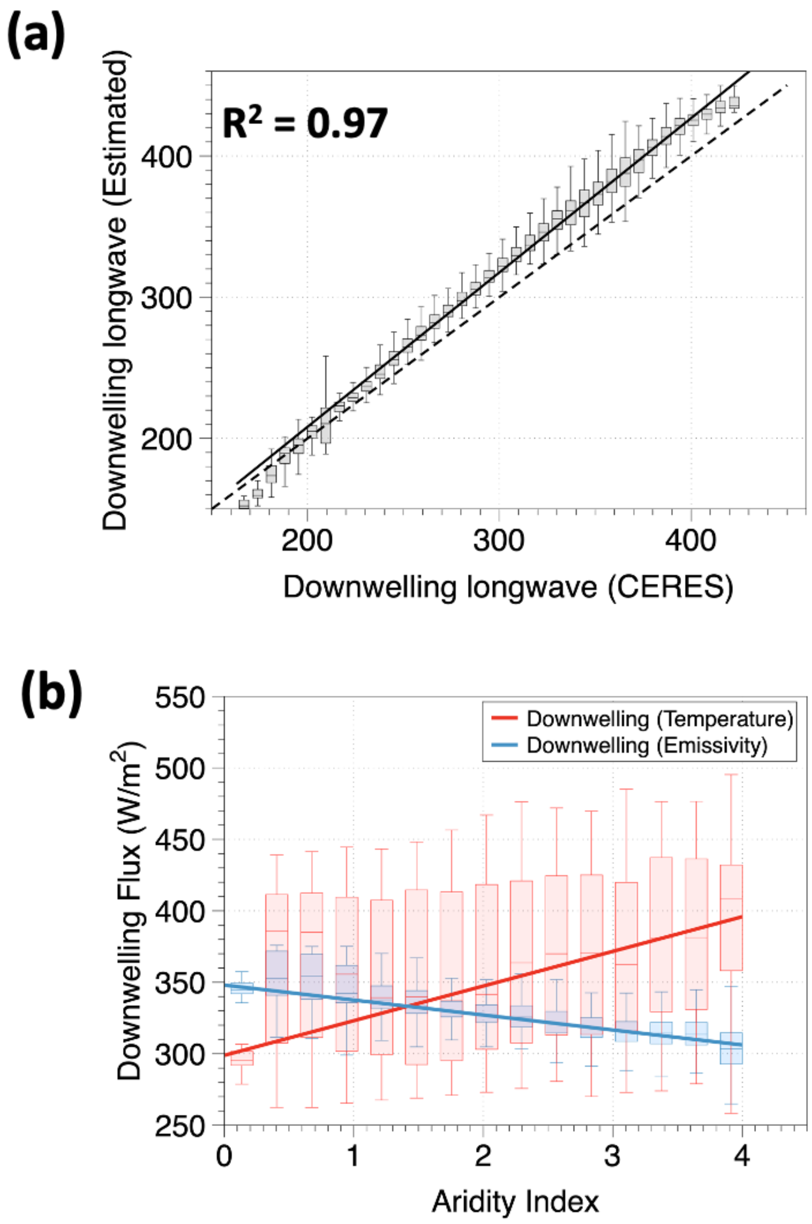

Figure S7: (a) Comparison of Downwelling longwave radiation estimated from the empirical formulation by (Brutsaert, 1975) and (Crawford & Duchon, 1999) with the downwelling flux from CERES-EBAF over global land. (b) Variation of decomposed downwelling longwave radiation into the atmospheric heating term (red) and emissivity term (blue) with Aridity index across the globe.

Figure S8

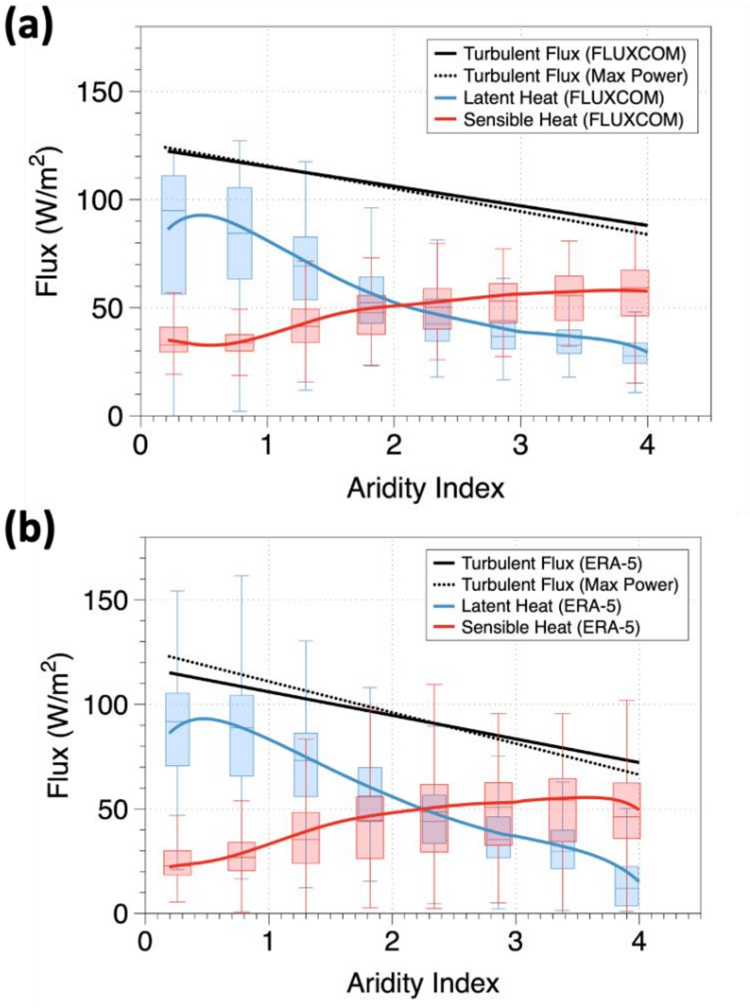

Figure S8: Same as figure 2e but for (a) FLUXCOM data and (b) ERA-5 data

Figure S9

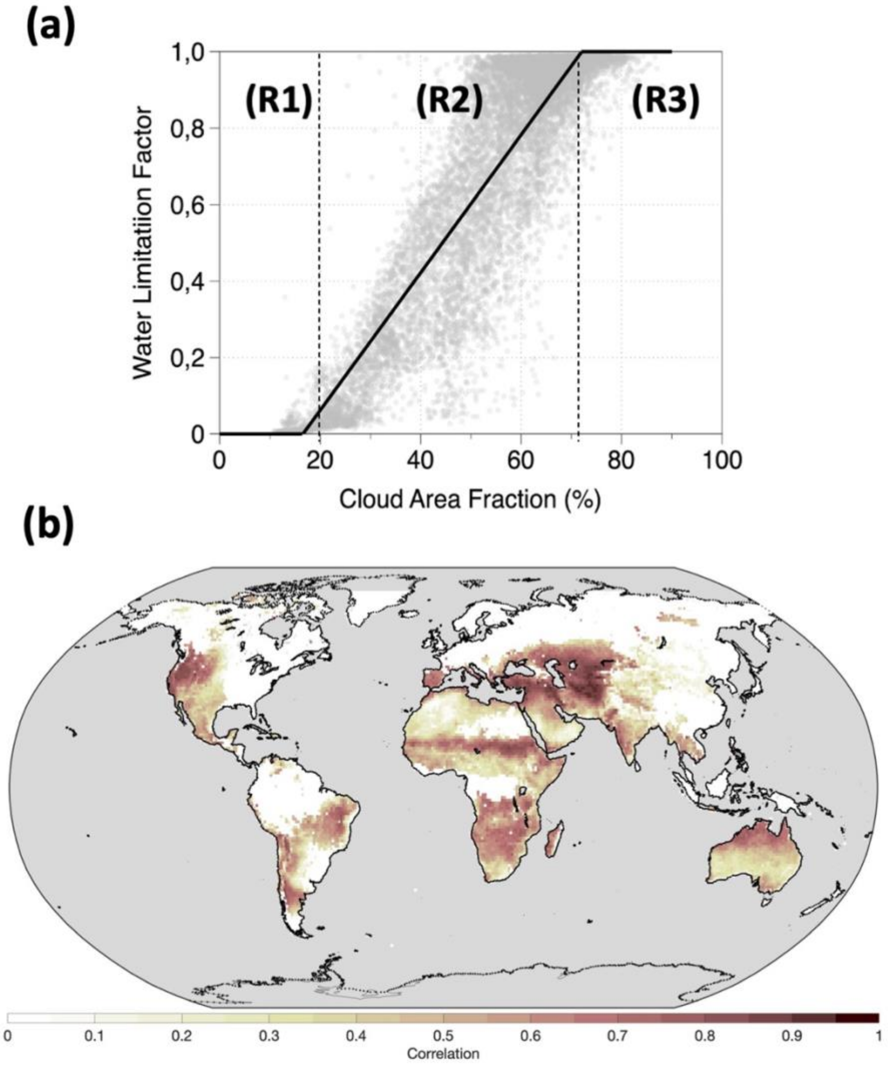

Figure S9: a) Variation of water limitation factor (calculated as the ratio of actual to potential evaporation) with cloud area fraction. (b) Global map of correlation between water limitation (calculated as the ratio of actual to potential evaporation from GLEAM) to cloud area fraction (from CERES) over global grids. Correlation was shown over only those grids which undergo at least a 20% change in water limitation throughout the year.

Table T1: List of all the variables used in the text with their notations, data source, and data reference

Notation	Variable Name	Dataset	Doi/Webpage
R_s	Absorbed Solar radiation at surface (W/m^2)	CERES EBAF 4.1, FLUXNET and ERA-5	10.5067/TERRA-AQUA/CERES/EBAF_L3B.004.1<br><br>https://fluxnet.org/data/fluxnet2015-dataset/<br><br>DOI: 10.24381/cds.f17050d7
R_{ld}	Downwelling longwave radiation at surface (W/m^2)		
$R_{l,up}$	Upwelling longwave radiation at surface (W/m^2)		
T_s	Surface Temperature (K), derived from $R_{l,up}$		
$R_{s,clear}$	Absorbed solar radiation at surface for clear-sky conditions (W/m^2)		
$R_{ld,clear}$	Downwelling longwave radiation at surface for clear-sky conditions (W/m^2)		
f_c	Cloud Area Fraction (%)		
$R_{s,toa}$	Net Solar radiation at top of atmosphere (W/m^2)		
$R_{l,toa}$	Outgoing longwave radiation at top of atmosphere (W/m^2)		
T_r	Radiative Temperature (K), derived from $R_{l,toa}$		
$\frac{dU}{dt}$	Seasonal heat storage (W/m^2)		
H	Sensible heat flux (W/m^2)	FLUXCOM and FLUXNET	https://www.fluxcom.org/EF-Download/<br>https://fluxnet.org/data/fluxnet2015-dataset/
LE	Latent heat flux (W/m^2)		
E_{act}	Actual Evaporation (mm/day)	GLEAM	https://www.gleam.eu/
E_{pot}	Potential Evaporation (mm/day)		
P	Precipitation (mm/day)	GPCP V1.3	10.7289/V5RX998Z

Table T2: List of all the sites from FLUXNET-2015 dataset used in the present study

SITE NAME	SITE ID	START YEAR	END YEAR	LATITUDE	LONGITUDE
Neustift	AT-Neu	2002	2012	47.1167	11.3175
Alice Springs	AU-ASM	2010	2014	-22.283	133.249
Adelaide River	AU-Ade	2007	2009	-13.0769	131.1178
Calperum	AU-Cpr	2010	2014	-34.0021	140.5891
Cumberland Plains	AU-Cum	2012	2014	-33.6133	150.7225
Daly River Savanna	AU-DaP	2007	2013	-14.0633	131.3181
Emerald Queensland	AU-Emr	2011	2013	-23.8587	148.4746
Great Western Woodlands WA	AU-GWW	2013	2014	-30.1913	120.6541
Loxton	AU-Lox	2008	2009	-34.4704	140.6551
Red Dirt Melon Farm NT	AU-RDF	2011	2013	-14.5636	132.4776
Riggs Creek	AU-Rig	2011	2014	-36.6499	145.5759
Robson Creek Queensland	AU-Rob	2014	2014	-17.1175	145.6301
Sturt Plains	AU-Stp	2008	2014	-17.1507	133.3502
Ti Tree East	AU-TTE	2012	2014	-22.287	133.64
Wallaby Creek	AU-Wac	2005	2008	-37.4259	145.1878
Whroo	AU-Whr	2011	2014	-36.6732	145.0294
Wombat	AU-Wom	2010	2014	-37.4222	144.0944
Jaxa	AU-Ync	2012	2014	-34.9893	146.2907
Brasschaat	BE-Bra	1996	2014	51.3092	4.5206
Lonzee	BE-Lon	2004	2014	50.5516	4.7461
Santarem-Km83-Logged Forest	BR-Sa3	2000	2004	-3.018	-54.9714
Ontario	CA-Gro	2003	2014	48.2167	-82.1556
Saskatchewan - Western Boreal forest burned in 1977	CA-SF1	2003	2006	54.485	-105.8176
Saskatchewan - Western Boreal forest burned in 1989	CA-SF2	2001	2005	54.2539	-105.8775
Saskatchewan - Western Boreal forest burned in 1998	CA-SF3	2001	2006	54.0916	-106.0053
Chamau	CH-Cha	2005	2014	47.2102	8.4104
Davos- Seehorn forest	CH-Dav	1997	2014	46.8153	9.8559
Früebüel	CH-Fru	2005	2014	47.1158	8.5378
Oensingen	CH-Oe1	2002	2008	47.2858	7.7319

grassland					
Changling	CN-Cng	2007	2010	44.5934	123.5092
Haibei Alpine Tibet site	CN-HaM	2002	2004	37.37	101.18
Bily Kriz forest	CZ-BK1	2004	2014	49.5021	18.5369
CZECHWET	CZ-wet	2006	2014	49.0247	14.7704
Anklam	DE-Akm	2009	2014	53.8662	13.6834
Gebesee	DE-Geb	2001	2014	51.1001	10.9143
Grillenburg	DE-Gri	2004	2014	50.9495	13.5125
Hainich	DE-Hai	2000	2012	51.0792	10.453
Klingenberg	DE-Kli	2004	2014	50.8929	13.5225
Lackenberg	DE-Lkb	2009	2013	49.0996	13.3047
Leinefelde	DE-Lnf	2002	2012	51.3282	10.3678
Oberbärenburg	DE-Obe	2008	2014	50.7836	13.7196
Rollesbroich	DE-RuR	2011	2014	50.6219	6.3041
Selhausen Juelich	DE-RuS	2011	2014	50.8659	6.4472
Schechenfilz Nord	DE-SfN	2012	2014	47.8064	11.3275
Spreewald	DE-Spw	2010	2014	51.8923	14.0337
Tharandt	DE-Tha	1996	2014	50.9636	13.5669
Zarnekow	DE-Zrk	2013	2014	53.8759	12.889
Zackenberg Fen	DK-ZaF	2008	2011	74.4814	-20.5545
Jokioinen	FI-Jok	2000	2003	60.8986	23.5135
Lettosuo	FI-Let	2009	2012	60.6418	23.9597
Lompolojänkkä	FI-Lom	2007	2009	67.9972	24.2092
Grignon	FR-Gri	2004	2014	48.8442	1.9519
Le Bray	FR-LBr	1996	2008	44.7171	-0.7693
Puechabon	FR-Pue	2000	2014	43.7414	3.5958
Ankasa	GH-Ank	2011	2014	5.2685	-2.6942
Castel d'Asso 1	IT-CA1	2011	2014	42.3804	12.0266
Castel d'Asso 2	IT-CA2	2011	2014	42.3772	12.026
Castel d'Asso 3	IT-CA3	2011	2014	42.38	12.0222
Collelongo- Selva Piana	IT-Col	1996	2014	41.8494	13.5881
Ispra ABC-IS	IT-Isp	2013	2014	45.8126	8.6336
Lavarone2	IT-La2	2000	2002	45.9542	11.2853
Lavarone	IT-Lav	2003	2014	45.9562	11.2813
Monte Bondone	IT-MBo	2003	2013	46.0147	11.0458
Arca di Noé - Le Prigionette	IT-Noe	2004	2014	40.6061	8.1515
Parco Ticino forest	IT-PT1	2002	2004	45.2009	9.061
Renon	IT-Ren	1998	2013	46.5869	11.4337

Roccarespampani 1	IT-Ro1	2000	2008	42.4081	11.93
Roccarespampani 2	IT-Ro2	2002	2012	42.3903	11.9209
San Rossore 2	IT-SR2	2013	2014	43.732	10.291
San Rossore	IT-SRo	1999	2012	43.7279	10.2844
Torgnon	IT-Tor	2008	2014	45.8444	7.5781
Moshiri Birch Forest Site	JP-MBF	2003	2005	44.3869	142.3186
Seto Mixed Forest Site	JP-SMF	2002	2006	35.2617	137.0788
Pasoh Forest Reserve (PSO)	MY-PSO	2003	2009	2.973	102.3062
Horstermeer	NL-Hor	2004	2011	52.2404	5.0713
Loobos	NL-Loo	1996	2014	52.1666	5.7436
Adventdalen	NO-Adv	2011	2014	78.186	15.923
Fyodorovskoye	RU-Fyo	1998	2014	56.4615	32.9221
Dahra	SN-Dhr	2010	2013	15.4028	-15.4322
ARM USDA UNL OSU Woodward Switchgrass 1	US-AR1	2009	2012	36.4267	-99.42
ARM USDA UNL OSU Woodward Switchgrass 2	US-AR2	2009	2012	36.6358	-99.5975
ARM Southern Great Plains site- Lamont	US-ARM	2003	2012	36.6058	-97.4888
GLEES Brooklyn Tower	US-GBT	1999	2006	41.3658	-106.2397
GLEES	US-GLE	2004	2014	41.3665	-106.2399
Goodwin Creek	US-Goo	2002	2006	34.2547	-89.8735
Fermi National Accelerator Laboratory- Batavia (Prairie site)	US-IB2	2004	2011	41.8406	-88.241
Ivotuk	US-Ivo	2004	2007	68.4865	-155.7503
Lost Creek	US-Los	2000	2014	46.0827	-89.9792
Metolius mature ponderosa pine	US-Me2	2002	2014	44.4523	-121.5574
Metolius-second young aged pine	US-Me3	2004	2009	44.3154	-121.6078
Metolius Young Pine Burn	US-Me6	2010	2014	44.3233	-121.6078
Niwot Ridge Forest (LTER NWT1)	US-NR1	1998	2014	40.0329	-105.5464
Olentangy River Wetland Research Park	US-ORv	2011	2011	40.0201	-83.0183
Poker Flat	US-Prr	2010	2014	65.1237	-147.4876

Research Range Black Spruce Forest					
Santa Rita Creosote	US-SRC	2008	2014	31.9083	-110.8395
Santa Rita Grassland	US-SRG	2008	2014	31.7894	-110.8277
Santa Rita Mesquite	US-SRM	2004	2014	31.8214	-110.8661
Sylvania Wilderness Area	US-Syv	2001	2014	46.242	-89.3477
Twitchell Wetland West Pond	US-Tw1	2012	2014	38.1074	-121.6469
Twitchell Corn	US-Tw2	2012	2013	38.1047	-121.6433
Twitchell Alfalfa	US-Tw3	2013	2014	38.1159	-121.6467
Twitchell East End Wetland	US-Tw4	2013	2014	38.103	-121.6414
UMBS Disturbance	US-UMd	2007	2014	45.5625	-84.6975
Vaira Ranch-Ione	US-Var	2000	2014	38.4133	-120.9507
Willow Creek	US-WCr	1999	2014	45.8059	-90.0799
Walnut Gulch Lucky Hills Shrub	US-Whs	2007	2014	31.7438	-110.0522
Walnut Gulch Kendall Grasslands	US-Wkg	2004	2014	31.7365	-109.9419
Skukuza	ZA-Kru	2000	2013	-25.0197	31.4969
Mongu	ZM-Mon	2000	2009	-15.4378	23.2528

SI References:

1. Kleidon, A., and M. Renner (2013), Thermodynamic limits of hydrologic cycling within the Earth system: Concepts, estimates, and implications, Hydrol. Earth Syst. Sci., 17(7), 2873– 2892, doi:10.5194/hess-17-2873-2013.

2. Dhara, C., Renner, M., and Kleidon, A.: Broad climatological variation of surface energy balance partitioning across land and ocean predicted from the maximum power limit, Geophys. Res. Lett., 43, 7686–7693, 2016

3. Kleidon, A. and Renner, M.: Diurnal land surface energy balance partitioning estimated from the thermodynamic limit of a cold heat engine, Earth Syst. Dynam., 9, 1127–1140, https://doi.org/10.5194/esd-9-1127-2018, 2018.

4. Kato, S., Rose, F. G., Rutan, D. A., Thorsen, T. J., Loeb, N. G., Doelling, D. R., Huang, X., Smith, W. L., Su, W., & Ham, S. (2018). Surface Irradiances of Edition 4.0 Clouds and the Earth's Radiant Energy System (CERES) Energy Balanced and Filled (EBAF) Data Product, Journal of Climate, 31(11), 4501-4527.

5. Loeb, N. G., D. R. Doelling, H. Wang, W. Su, C. Nguyen, J. G. Corbett, L. Liang, C. Mitrescu, F. G. Rose, and S. Kato, 2018: Clouds and the Earth's Radiant Energy System (CERES) Energy Balanced and Filled (EBAF) Top-of-Atmosphere (TOA) Edition-4.0 Data Product. J. Climate, 31, 895-918, doi: 10.1175/JCLI-D-17-0208.1.

6. Slayter, R. O. and McIlroy, I. C.: Practical Micrometeorology, CSIRO, Melbourne, Australia, 310 pp., 1961.

7. Priestley, C. H. B., & Taylor, R. J. (1972). On the assessment of surface heat flux and evaporation using large-scale parameters. Monthly Weather Review, 100, 81– 92. https://doi.org/10.1175/1520-0493(1972)100<0081:OTAOSH>2.3.CO;2

8. B. Martens, D.G. Miralles, H. Lievens, R. van der Schalie, R.A.M. de Jeu, D. Fernández-Prieto, H.E. Beck, W.A. Dorigo, N.E.C. Verhoest, GLEAM v3: satellite- based land evaporation and root-zone soil moisture, Geosci. Model Dev. 10 (2017) 1903–1925, https://doi.org/10.5194/gmd-10-1903-2017.

9. Brutsaert, W., 1975: On a derivable formula for long-wave radiation from clear skies. Water Resources Research, 11, 742-744.

10. Crawford, T. M., and C. E. Duchon, 1999: An Improved Parameterization for Estimating Effective Atmospheric Emissivity for Use in Calculating Daytime Downwelling Longwave Radiation. Journal of Applied Meteorology, 38, 474-480.
